# CT Radiomics for the Preoperative Prediction of Ki67 Index in Gastrointestinal Stromal Tumors: A Multi-Center Study

**DOI:** 10.3389/fonc.2021.689136

**Published:** 2021-09-14

**Authors:** Yilei Zhao, Meibao Feng, Minhong Wang, Liang Zhang, Meirong Li, Chencui Huang

**Affiliations:** ^1^First Affiliated Hospital, School of Medicine, Zhejiang University, Hangzhou, China; ^2^First Affiliated Hospital of Wannan Medical College, Wuhu, China; ^3^Zhejiang Cancer Hospital, University of Chinese Academy of Sciences, Hangzhou, China; ^4^Beijing Deepwise & League of PHD Technology Co., Ltd, Beijing, China

**Keywords:** gastrointestinal stromal tumors (GIST), ki67, radiomics, computed-tomography (CT), prognostic

## Abstract

**Purpose:**

This study established and verified a radiomics model for the preoperative prediction of the Ki67 index of gastrointestinal stromal tumors (GISTs).

**Materials and Methods:**

A total of 344 patients with GISTs from three hospitals were divided into a training set and an external validation set. The tumor region of interest was delineated based on enhanced computed-tomography (CT) images to extract radiomic features. The Boruta algorithm was used for dimensionality reduction of the features, and the random forest algorithm was used to construct the model for radiomics prediction of the Ki67 index. The receiver operating characteristic (ROC) curve was used to evaluate the model’s performance and generalization ability.

**Results:**

After dimensionality reduction, a feature subset having 21 radiomics features was generated. The generated radiomics model had an the area under curve (AUC) value of 0.835 (95% confidence interval(CI): 0.761–0.908) in the training set and 0.784 (95% CI: 0.691–0.874) in the external validation cohort.

**Conclusion:**

The radiomics model of this study had the potential to predict the Ki67 index of GISTs preoperatively.

## Introduction

Gastrointestinal stromal tumors (GISTs) are the most common mesenchymal-derived tumors of the gastrointestinal tract, with complex biological behaviors. Recurrence and metastasis of GISTs are the main factors associated with patient survival. The pathogenesis of GISTs is mainly due to functional gene mutations of *KIT*, which lead to the continuous activation of the proto-oncogene receptor tyrosine kinase (1). The molecular targeted drug imatinib mesylate blocks tyrosine kinase-activated pathways and is often used to control postoperative recurrence of high-risk GISTs and in neoadjuvant therapy (2, 3).

The GISTs risk stratification criteria issued by the National Institution of Health is the most commonly used GISTs biological behavior and clinical outcome evaluation criteria (4). The mitosis count of the specimen is a significant factor in these criteria. However, the identification of mitosis count depends on the pathologists’ skills and experience, resulting in a problem of reproducibility of findings and affecting the risk stratification accuracy. Ki67 is a nuclear antigen related to proliferating cells and is a marker of cell division and proliferation activity. Its expression level correlates with the degree of tumor cell proliferation. Tumors with active proliferation are susceptible to invasion and distant metastasis (5, 6). The Ki67 index is evaluated by immunohistochemistry. In Ki67 immunohistochemistry, cell nuclei in proliferating cells are stained positively, while the nuclei in nonproliferating cells remain negative. Ki67 immunohistochemistry eliminates the bias in morphological observations and results in a consistent interpretation from different pathologists (7, 8). Second, Ki67 is expressed in all phases of the cell cycle (i.e., G1, S, G2, and M phases), whereas the mitosis is only present in the M phase. Ki67 index is more comprehensive and consistent compared with the mitosis count (9).

In the era of precision therapy for GISTs, the accurate prognosis stratification for patients suffering from GISTs is of great importance. Studies have found that patients with Ki67 index > 8% have a high risk of recurrence, which is an adverse factor for adjuvant imatinib therapy. Ki67 index can also play as an assistant in the stratified diagnosis and treatment among GISTs patients. The CT imaging characteristics, including size, contour, and tumor margin, are correlated with the Ki67 index in GISTs, suggesting that imaging features can be used to predict the expression of Ki67 in GISTs (9). Hence, in this study we constructed a prediction model of the Ki67 index (cutoff value of 8%) for patients with GISTs by extracting the texture and morphological features of tumors from enhanced CT images, and verified the model with independent external data to evaluate the generalization ability.

## Materials and Methods

### Patients

This retrospective study was approved by the Institutional Review Board of the First Affiliated Hospital Zhejiang University School of Medicine (Zhejiang, China). The signed informed consent forms were waived. This study included patients with GISTs in three hospitals from January 1, 2016, to July 1, 2020. The inclusion criteria of this study were as follows (1) GIST was diagnosed pathologically; (2) patient had enhanced CT examination within 15 days before surgery; and (3) the Ki67 index was reported in the pathological results. The exclusion criteria were patients who received neoadjuvant treatment with imatinib or other tyrosine kinase inhibitors before CT examination.

The Ki67 index was evaluated by immunohistochemistry within 7 days after surgical. The patients were divided into two groups based on the cutoff value of 8% for the Ki67 index: high-Ki67 group (Ki67 index >8%) and low-Ki67 group (Ki67 index ≤8%).

According to the above criteria, 344 patients with GISTs were included in this study. Data from the First Affiliated Hospital Zhejiang University School of Medicine (Zhejiang, China) were placed in the training set, and the data from the other two hospitals were placed in the external validation set. Descriptive statistical analyses were performed on the clinical data of the training and validation sets, where a t-test was used for continuous variables and a chi-square test was used for categorical variables. All statistical analyses were performed using R software (version 3.4.1; http://www.rproject.org). P < 0.05 (two-sided) was considered statistically significant.

### CT Technology

The examination equipment included the Brilliance 64-slice spiral CT and the Brilliance 256-slice spiral CT scanners (Philips Healthcare), and the SOMATOM Definition dual-source CT scanner (Siemens Healthineers). The CT scanning range was from the diaphragm dome to the lower margin of the pubic symphysis and the scanning direction was from the cranial to caudal. The patients were required to hold their breath during the CT. The scanning parameters of the Philips’ CT scanners were 64 × 0.625 mm collimation, 250 mm field of view (FOV), 120 kV tube voltage, 250 mA tube current, 5 mm slice thickness, 5 mm slice interval, 0.4 s tube rotation time, 0.891 pitch, standard (B) algorithm, and 512 × 512 matrix. The scanning parameters of the Siemens’ CT scanner were 128 × 0.6 mm collimation, 50 min FOV, 120 kV tube voltage, 200 mA tube current, 0.5 s tube rotation time, 0.6 pitch, standard (B) algorithm, and 512 × 512 matrix. A high-pressure syringe was used to inject 70–100 mL of contrast agent into the patient’s antecubital vein at a flow rate of 2.5–3.5 mL/s. A plain scan, arterial phase scan at 25–30 s after contrast agent injection, and venous phase scan at 55–60 s after contrast agent injection were performed.

### Image Segmentation and Feature Extraction

**Figure 1:**
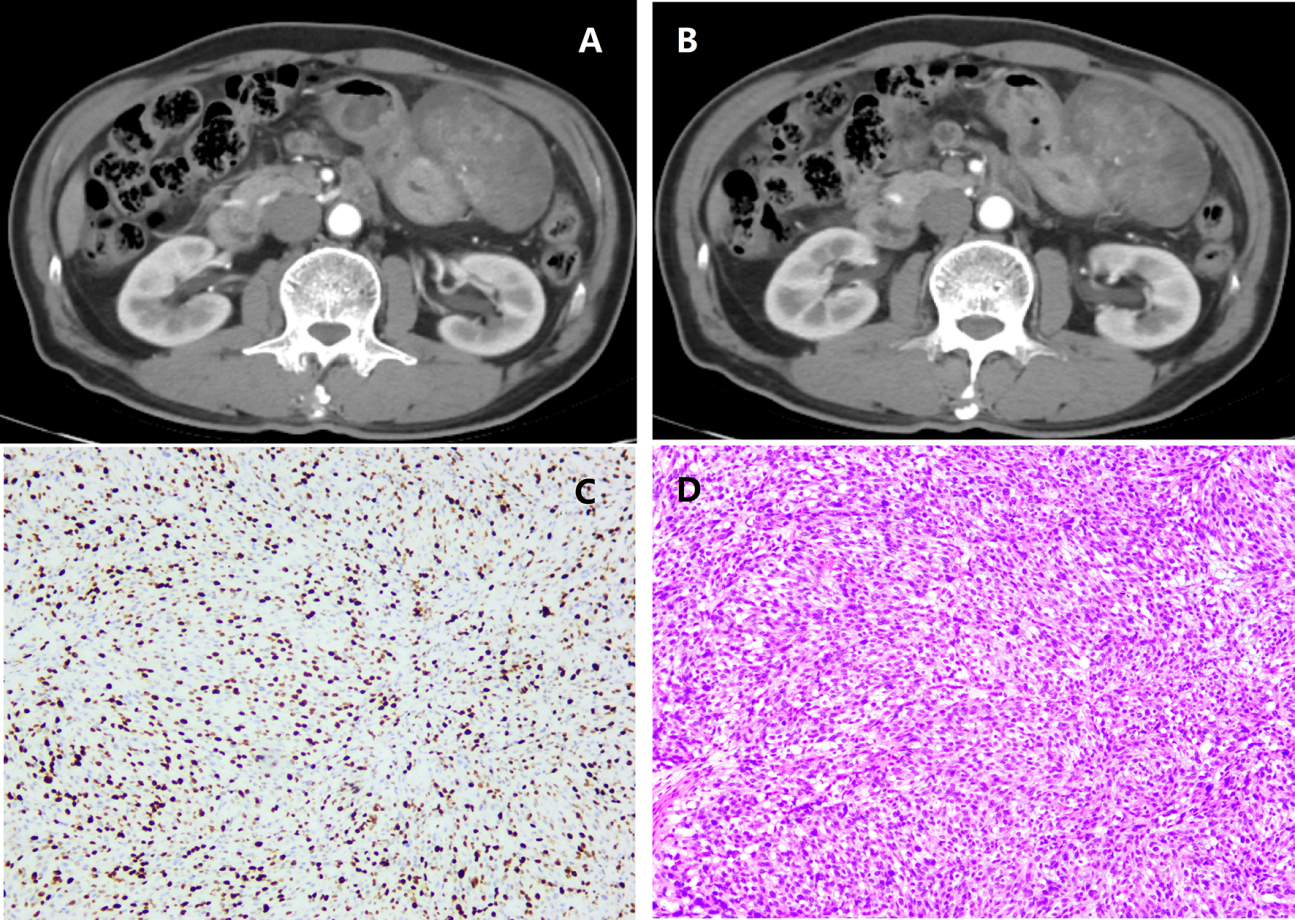
A patient with intestinal gastrointestinal stromal tumor (GIST) belonging to the high malignant potential. **(A)** Arterial phase; **(B)** Venous phase; **(C)** Immunohistochemical photomicrograph showed a high Ki67 index (about 35%, original magnification, ×10); **(D)** Pathology demonstrated a high-risk GIST with a high mitotic rate (> 4/HPF, original magnification, ×20).

Tumor segmentation and radiomics feature calculation were based on MATLAB’s IBEX software package (10). On the arterial and venous phase CT images before the GIST surgery, the region of interest (ROI) was delineated in layers along the tumor contour’s edge (**Figure 1**). ROI segmentation was performed by a radiologist and confirmed by another radiologist. The two radiologists were blinded to the Ki67 index before ROI segmentation.

Before feature extraction, all images were resampled according to the voxel size of 1×1×1 mm^3^, and the gray-level value was normalized to 1–64 for preprocessing. The radiomics parameters used in this study included six categories, i.e., histogram parameter (n = 48), 2.5D and 3D gray-level co-occurrence matrix (n = 594), neighborhood gray-tone difference matrix (n = 10), gray-level run-length matrix (n = 34), and shape and size (n = 18). A total of 704 radiomics parameters were extracted from the lesions in each phase, so there were 1,408 radiomics parameters for each patient.

### Dimensionality Reduction and Modeling

The radiomics features were first subjected to Spearman’s correlation analysis, with a correlation coefficient threshold of 0.8. Subsequently, the Boruta algorithm was used to perform dimensionality reduction again. The random forest classifier was used to construct a prediction model, perform 10-fold cross-validation on the training set, and eventually evaluate the model’s generalization ability in the external validation set. The performance of the model was evaluated on the basis of receiver operating characteristic (ROC) curves. Delong test was used to evaluate the AUC values of the model in the training set and external validation set. Then, the Brier score was used to quantify overall performance of the model. Brier score is the mean squared difference between the observed and predicted outcome. It is a combination of calibration and differentiation.

## Results

The clinical characteristics of the patients with GISTs (**Table 1**) indicated no statistical differences in age, gender, and tumor location between the high-Ki67 group and the low-Ki67 group in the training or validation sets. In addition, no significant difference in the clinical characteristics was found between the training and external validation sets.

**Table 1:** Patient characteristics in the training and external validation cohorts.

	Training cohort (N=192)			External validation cohort (N=152)		
	Low-Ki67	High-Ki67	P	Low-Ki67	High-Ki67	P
	(N=128)	(N=64)		(N=107)	(N=45)	
Age (years)	61.8 ± 11.23	63.02 ± 12.82	0.536	55.86 ± 10.74	60.78± 13.29	0.237
Sex (n)			0.609			0.305
Female	61	28		59	27	
Male	67	36		48	18	
Site (n)			0.459			0.286
Gastric	83	26		31	15	
Intestinal	45	38		76	30	

P < 0.05 indicates that difference is statistically significant.

**Figure 2:**
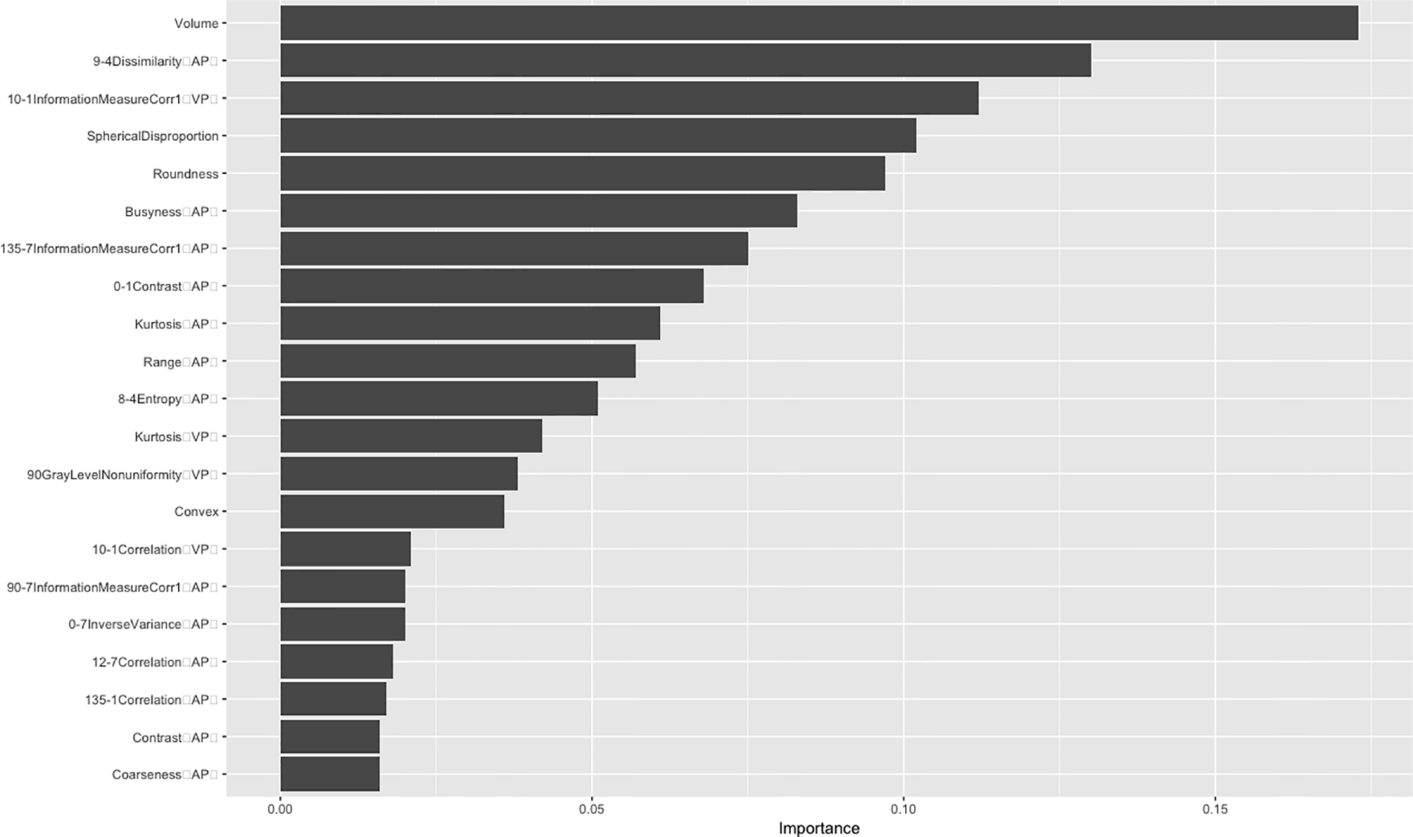
The importance of radiomics features. AP, arterial phase; VP, venous phase.

**Figure 3:**
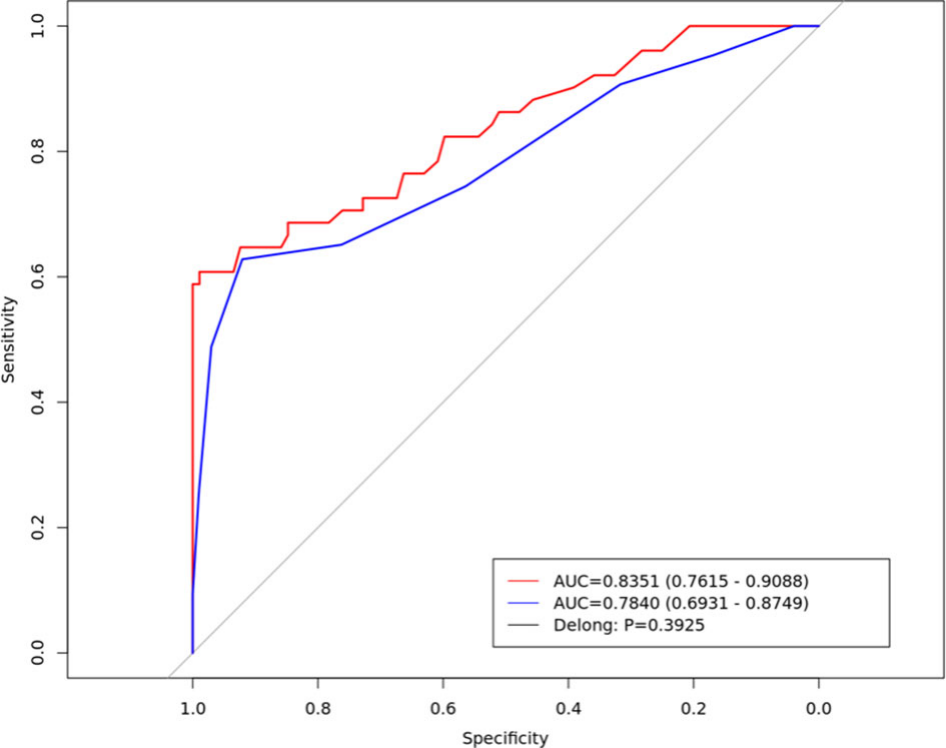
The Receiver operating characteristic curve of the radiomics prediction model in the training set (red line) and external validation set (blue line).

After dimensionality reduction, 21 radiomics features were identified, Among these, seven gray-level co-occurrence matrices features (3D), four shape and size features, three gray-level co-occurrence matrices features (2.5D), three neighborhood gray-tone difference matrix features, three histogram features and only one gray level run length matrix were selected. There were 13 features in the venous phase and four in the arterial phase (**Figure 2**). The ROC curve was used to evaluate the performance of the model. The AUC values of the training set and external validation set were 0.835 (95% confidence interval(CI): 0.761–0.908) and 0.784 (95% CI: 0.691–0.874), respectively (**Figure 3**). Brier score of the model was 0.14 in the training set and 0.16 in the external validation set. DeLong test has showed the prediction model had no significantly reduced efficiency in the external validation set.

## Discussion

Based on CT images, the radiomics model’s performance in preoperative prediction of the Ki67 index was studied. Through independent external validation of multiple centers, this study show that the model performed well in predicting whether the Ki67 index was low or high and had good generalization ability.

With the extensive application of adjuvant imatinib therapy, studies have evaluated the correlation between the Ki67 index and adjuvant imatinib therapy. Kaplan-Meier survival analysis and multivariate analysis showed that patients with GISTs and a Ki67 index >8% had a worse prognosis, which was related to imatinib treatment. Particularly, in the high-risk GIST group the rate of recurrence was higher in patients with a Ki67 index >8% (11), suggesting that the Ki67 index >8% was an unfavorable factor for adjuvant imatinib therapy (12, 13). And Sugita et al. have found that 8% cutoff was a good supplementary prognostic factor for NIH risk stratification. Ki67 greater than 8% would indicate poor prognosis in both low - and intermediate-risk groups (14). Raut et al. also concluded that Ki67 was one of the reference markers for imatinib’s adjuvant treatment time after surgery (15). High Ki67 expression is associated with poor pathological characteristics and aggressive behaviors of GISTs and can be used as an independent prognostic marker of GISTs (16), But in the existing GIST diagnostic and treatment systems, although it is unrealistic to directly use the Ki67 index to replace the NIH risk stratification of GISTs, the Ki67 index (cutoff value of 8%) can become a supplement to the NIH risk stratification standard for evaluating the prognosis of high-risk GISTs and selecting a suitable population for adjuvant imatinib therapy. CT has the advantages of being noninvasive, objective, and convenient. Preoperative radiomics prediction of the Ki67 index (cutoff value of 8%) provided an additional value for evaluating the prognosis of GISTs.

Numerous studies have demonstrated a close relationship between the imaging characteristics of a tumor and the Ki67 index. The mean, median, and percentage of the apparent diffusion coefficient in functional diffusion imaging of hepatocellular carcinoma are significantly and negatively correlated with the Ki67 level (17). A study by Peng et al. established a multiple linear regression model for preoperative prediction of the Ki67 index of pulmonary nodules with ground-glass opacity based on computed tomography (CT) images (18). Previous studies on GIST imaging for predicting the Ki67 index had two cutoff values. A study by Li et al. used 5% as the cutoff value of the Ki67 index. Through logistic regression analysis, tumor size and ulcers’ presence were the most effective features to predict the Ki67 index being ≤5% or >5% in GISTs (19). A study by Zhang et al. set the cutoff value of the Ki67 index to 10% and used radiomics based on enhanced CT images to predict the Ki67 index of patients with GISTs. Their radiomics model performed well, and the AUC of the training set reached 0.801 (20).

NIH risk stratification has showed certain deficiencies with the extensive application of targeted therapies; and it has been removed from the National Comprehensive Cancer Network guidelines since 2015 (21). As a supplementary factor, Ki67 (8%) may make NIH risk stratification better, and its high consistency makes it possible to replace the counting of mitosis count and integrate with other parameters to be a new prognostic stratification (14, 22). Currently, most radiology studies of the GISTs are still focusing on NIH stratification, while those for Ki67 are relatively few. Our results were consistent with those of two previous similar studies, both suggesting that imaging could predict the Ki67 index level, and both found that tumor size played a very important role in Ki67 prediction. Ki67 is related to mitosis in cells, so the higher the index, the more active the tumor proliferation, resulting in a larger tumor size of GISTs (23, 24). Notably, most of the previous radiomics studies of GISTs were mainly single-center studies. The different scanning equipment, parameter settings, and post-processing reconstruction algorithms led to differences in radiomics parameters (25–27). The heterogeneity of single-center imaging data is low, and many prediction models have not been externally validated, so the possibility of overfitting is high (28). Although the prediction model’s performance in our study lower in the external validation set, it still maintained at 0.75. Delong test indicating that the model kept a reliable generalization ability.

In the post-NIH stratification era, the Ki67 imaging prediction research has certain clinical significance and great potential. This study has some limitations. First, it is a retrospective study with selection bias; further prospective studies are needed to verify our radiomics model. Second, although this was a multi-center study, we only collected data from three hospitals, and our sample size is small; further studies with a larger sample size with data collected from more hospitals will be necessary. Third, no uniform standard for selecting the cutoff value of the Ki67 index is available, and the cutoff values of the Ki67 index are still in dispute.

## Conclusion

In summary, the Ki67 index (cutoff value of 8%) can assist NIH risk classification of GISTs, providing supplementary information for the prognostic evaluation of GISTs. As a noninvasive and objective examination, CT radiomics effectively predicted the Ki67 index of GISTs and has the potential for clinical translation. However, verification using multi-center, large sample data will be required before the radiomics model can be truly applied in clinical practices.

## Data Availability Statement

The raw/processed data required to reproduce these findings cannot be shared at this time as the data also forms part of an ongoing study. Requests to access the datasets should be directed to gerxyuan@zju.edu.cn.

## Ethics Statement

The studies involving human participants were reviewed and approved by Institutional Review Board of the First Affiliated Hospital Zhejiang University School of Medicine. Written informed consent for participation was not required for this study in accordance with the national legislation and the institutional requirements.

## Author Contributions

YZ and ML: conception and design, writing, review, and revision of the manuscript. MW, LZ, CH and MF: analysis data. YZ: study supervision. All authors contributed to the article and approved the submitted version.

## Conflict of Interest

Author CH was employed by the company Deepwise & League of PHD Technology Co., Ltd.

The remaining authors declare that the research was conducted in the absence of any commercial or financial relationships that could be construed as a potential conflict of interest.

## Publisher’s Note

All claims expressed in this article are solely those of the authors and do not necessarily represent those of their affiliated organizations, or those of the publisher, the editors and the reviewers. Any product that may be evaluated in this article, or claim that may be made by its manufacturer, is not guaranteed or endorsed by the publisher.
